# The Biological Significance and Regulatory Mechanism of c-Myc Binding Protein 1 (MBP-1)

**DOI:** 10.3390/ijms19123868

**Published:** 2018-12-04

**Authors:** Zijin Liu, Aileen Zhang, Lamei Zheng, Abou-Fadel Johnathan, Jun Zhang, Genfa Zhang

**Affiliations:** 1 Beijing Key Laboratory of Gene Resource and Molecular Development/College of Life Sciences, Beijing Normal University, Beijing 100875, China; liuzijin1012@163.com (Z.L.); abz0010@berkeley.edu (A.Z.); zhenglm100@163.com (L.Z.); 2 Department of Integrative Biology, University of California, Berkeley, CA 94720-3200, USA; 3 Department of Biomedical Sciences, Texas Tech University Health Sciences Center EI Paso, EI Paso, TX 79905, USA; Johnathan.Abou-Fadel@ttuhsc.edu (A.-F.J.); jun.zhang2000@gmail.com (J.Z.)

**Keywords:** MBP-1, *ENO*, regulatory network, alternative translated protein, biological function

## Abstract

Alternatively translated from the *ENO* gene and expressed in an array of vertebrate and plant tissues, c-Myc binding protein 1 (MBP-1) participates in the regulation of growth in organisms, their development and their environmental responses. As a transcriptional repressor of multiple proto-oncogenes, vertebrate MBP-1 interacts with other cellular factors to attenuate the proliferation and metastasis of lung, breast, esophageal, gastric, bone, prostrate, colorectal, and cervical cancer cells. Due to its tumor-suppressive property, MBP-1 and its downstream targets have been investigated as potential prognostic markers and therapeutic targets for various cancers. In plants, MBP-1 plays an integral role in regulating growth and development, fertility and abiotic stress responses. A better understanding of the functions and regulatory factors of MBP-1 in plants may advance current efforts to maximize plant resistance against drought, high salinity, low temperature, and oxidative stress, thus optimizing land use and crop yields. In this review article, we summarize the research advances in biological functions and mechanistic pathways underlying MBP-1, describe our current knowledge of the *ENO* product and propose future research directions on vertebrate health as well as plant growth, development and abiotic stress responses.

## 1. Introduction

Widely distributed and highly conserved in vertebrates and plants, the *ENO* gene family encodes enolase (ENO), a glycolytic metalloenzyme that catalyzes the dehydration of 2-phospho-d-glycerate (2-PGA) to phosphoenolpyruvate (PEP) and c-Myc binding protein 1 (MBP-1), a transcription factor involved in cellular division, growth and response pathways [1]. In plants, *ENO* genes are referred to as low expression of osmotically responsive (*LOS*) genes [2]. Multiple ENO isoforms currently exist: vertebrate *ENO* encodes isoforms α-enolase (α-ENO), β-enolase (β-ENO), γ-enolase (γ-ENO) and enolase 4 (ENO4), while plant *LOS* encodes isoforms enolase 1 (ENO1), enolase 2 (ENO2) and enolase 3 (ENO3). Multiple isoforms have emerged due to the occurrence of two duplication events in the genomic evolution of the *ENO* family [1].

While mammalian β-ENO resides in muscle tissues, γ-ENO localizes in neuronal and neuroendocrine tissues and ENO4 exists in the testes; α-ENO is found in most adult tissues [3,4,5]. Thus far, most research studies have focused on the multiple functions and pathological significance of the α-ENO isoform. While α-ENO catalyzes glycolytic and gluconeogenic pathways in the cytoplasm, α-ENO may also bind to microtubule proteins and function as a plasminogen receptor on the plasma membrane [6]. The myriad functions of α-ENO have thus implicated the protein in numerous pathophysiological conditions—including cancer, parasitic infection, autoimmune disease and muscle generation [7,8,9,10,11,12]. Between vertebrate and plant enolase isoforms, vertebrate α-ENO and plant ENO2 maintain a high level of homology. Indeed, human α-ENO and *Arabidopsis thaliana* ENO2 share 72% similarity at the amino acid level [13]. In plants, the ENO2 isoform participates in an array of biological processes by playing an integral role in the growth, reproductive development, low temperature, drought and high salt stress responses of plants [14,15,16].

While the ubiquitous conserved nature and diverse functions of α-ENO and ENO2, encoded by *ENO1* and *LOS2,* respectively, have garnered the interest of many health and plant science researchers, *ENO1* and *LOS2* mRNA transcripts also generate the transcription factor c-Myc binding protein (MBP-1) by alternative translation [17]. In vertebrates, MBP-1 suppresses tumorigenesis and regulates the growth and metastasis of cancer cells by regulating genes expression, such as c-MYC, *COX-2* and *ERBB2*. Arabidopsis plants that the overexpression of *AtMBP-1* are hypersensitive to abscisic acid (ABA) during seed germination and show defects in vegetative growth and lateral stem development. Given the pathophysiological and agricultural significance of MBP-1, this article will review the biological functions and mechanistic foundation, and suggest possible future research directions, of vertebrate and plant MBP-1.

## 2. Basic Characteristics of MBP-1

**Figure 1:**
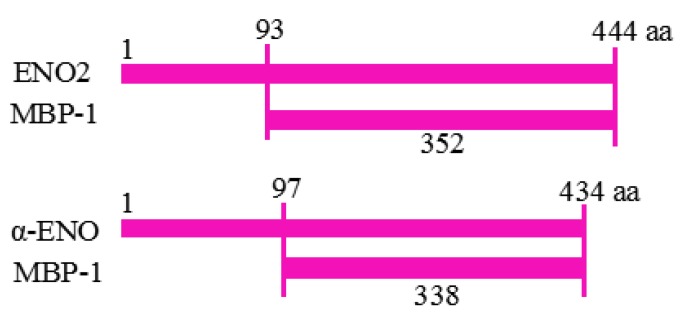
Diagram of ENO2/α-ENO protein and the truncated form MBP-1. aa indicates amino acid.

First identified by Ray and Miller in human cervical cancer cells (HeLa) cDNA library, vertebrate MBP-1 primarily localizes in the nucleus and functions as a transcription factor [18]. Human α-ENO and MBP-1, both encoded by *ENO1*, map to the short arm of chromosome 1 (1p35-p36). The translation initiation site (TIS) of MBP-1 is positioned 289 bp downstream of the α-ENO TIS [7,19]. As presented in Figure 1, vertebrate α-ENO/plant ENO2 are 434 and 444 amino acids in length, respectively, while vertebrate MBP-1/plant MBP-1 are 338 and 352 amino acids in length, respectively [7,13]. Although α-ENO cDNA and MBP-1 cDNA actually exhibit 97% homology, the truncated nature of MBP-1 results in differences in molecular weight, structure, subcellular localization, enzymatic activity and DNA-binding ability between the two *ENO1* gene products [17,20]. While ~48 kDa α-ENO/ENO2 localizes in the cytoplasm and plasma membrane, functioning as a glycolytic enzyme and plasminogen receptor, ~37 kDa MBP-1 localizes in the nucleus and lacks enzymatic activity. The truncated *ENO1*/*LOS2* product, instead, directly binds to DNA as well as other cellular partners and functions as a transcription factor [7,18]. A recent study also found that plant MBP-1 may reduce ENO2 transcription by inhibiting *LOS2* promoter activity, suggesting a feedback regulatory mechanism of ENO2 by MBP-1 [14]. The mechanism by which MBP-1 enters the nucleus and α-ENO reaches the plasma membrane, as well as how MBP-1 regulates ENO2 expression, has yet to be elucidated.

**Figure 2:**
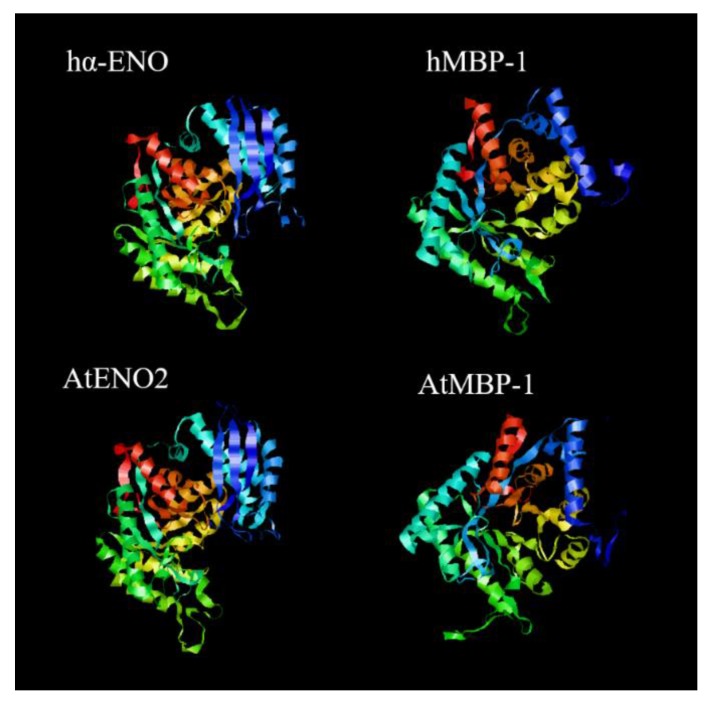
Predicted model of human α-ENO and MBP-1 (hα-ENO and hMBP-1) and *Arabidopsis thaliana* ENO2 and MBP-1 (AtENO2 and AtMBP-1). Blue marks N-terminus and red marks C-terminus. Other colors represent the different domains.

A majority of MBP-1 studies have focused on the influence of vertebrate MBP-1 in human pathophysiology and of plant MBP-1 in *Arabidopsis thaliana* growth and development, fertility and abiotic stress responses. Thus, the structures of human and *Arabidopsis thaliana* MBP-1 were simulated and visualized to compare vertebrate/plant α-ENO/MBP-1 in this review (Figure 2). Human α-ENO/MBP-1 and AtENO1/MBP-1 amino acid sequences were provided by Universal Protein Resource (UniProt) and secondary structures were predicted and visualized with Protein Homology/Analogy Recognition Engine 2.0 (Phyre2) and RasMol, respectively. The structural differences between α-ENO and MBP-1, specifically the distinct N-terminals of the two proteins, may account for the non-enzymatic nature and nuclear subcellular localization of MBP-1. The UniProt accession numbers, protein data bank (PDB) identifiers of the templates and associated sequence identities are provided in the Supplementary materials.

In an α-ENO monomer, the C-terminal contains an eightfold alpha/beta barrel structure. Larger and more evolutionarily stable than the N-terminal, the C-terminal exhibits β/β/α/α (β/α)_6_ topology and thus consists of two β-sheets, two α helices and an alternating β-sheet/ α-helices barrel. The N-terminal presents β_3_α_4_ topology, containing three β-strands and four α-helices. In enzymatically active dimers, α-ENO binds to α-ENO, β-ENO and γ-ENO in an antiparallel manner [1,7]. While the C-terminal of MBP-1 shares identical topology with the α-ENO C-terminal and contains a hydrophobic sequence (240–290 aa), the N-terminal of MBP-1 only consists of four α-helices and lacks the three β strands of the α-ENO N-terminal. The MBP-1 N-terminal also contains a proline-rich region [21].

## 3. MBP-1 in the Normal Tissues and Cancer Cells of Vertebrates

Despite being encoded by the same gene in vertebrates, endogenous levels of α-ENO are often higher than that of MBP-1 due to differences in translational efficiency, translational regulation and post-translational stability. Post-translational quantities of MBP-1 may also be impacted by MBP-1 transcript levels. *MBP-1* transcript has a translation efficiency 17.8-fold higher for MBP-1 than that of the *ENO1* transcript by vitro-coupled transcription and translation assay in plasmid. Under the normal condition, the transcript level is more than 100-fold lower than that of ENO1 in a variety of normal and cancer cells such as NHBE, BEAS-2B and A549 [22]. Subramanian et al. [23] also detected only very low levels of the endogenous MBP-1 transcript in HeLa cells. Moreover, MBP-1 is expressed in many normal tissues. In contrast to the ENO1 transcript, MBP-1 transcript is highly expressed in brain, liver and kidney tissues, and the MBP-1 mRNA is exclusively highly abundant in spleen, and was rarely found in stomach and colon tissues [22]. However, endogenous MBP-1 protein has not been detected in the cell lines examined so far, such as A549, HeLa, CL1-5 and breast cancer cells [22,24]. As compared to the MBP-1 TIS, the α-ENO TIS is preferentially optimized for translational apparatus assembly and function. Furthermore, the translation of MBP-1 mRNA is heavily regulated from the 5’-UTR and α-ENO transcript. In addition to differences at the transcriptional and translational level, α-ENO and MBP-1 demonstrate distinct post-translational stabilities. Since MBP-1 is subject to ubiquitin-mediated proteasomal degradation, MBP-1 maintains a half-life of approximately 2.5 h in normal COS-7 cells and thus exhibits a high endogenous turnover rate. The proteasomal inhibitor MG132 enhances MBP-1 stability [22].

## 4. MBP-1 in Tumorigenesis

While α-ENO and MBP-1 are found in a wide range of tissues and cell types, α-ENO and MBP-1 expression levels are differentially regulated in normal tissues. α-ENO mRNA is highly expressed in normal liver, brain and kidney tissues. Several studies revealed that as a potent plasminogen-binding protein, α-ENO is expressed in a variety of cell surfaces (T cells, B cells and neurons) and is also related to the pathogenic effects of many diseases, including cancer, Alzheimer’s disease and rheumatoid arthritis [25]; α-ENO can also affect the physiological metabolism, apoptosis and migration of cancer cells and is expected to be a novel pharmacological target or immunotherapeutic strategy for cancer treatment [10,26]. Similar to α-ENO, MBP-1 also demonstrates a significant influence on the growth, progression, and metastasis of cancer (Table 1). In the gastric cancer cell line SC-M1, the overexpression of MBP-1 suppresses the cell colony formation, migration and invasion [27,28]. In addition, MBP-1 can suppress the tumorigenicity of two breast cancer cell lines, MDA-MB-231 and MCF-7, by inhibiting anchorage-independent growth and invasive activity. The exogenous expression of MBP-1 also results in cell death and DNA fragmentation [29]. Lo et al. analyzed α-ENO and MBP-1 expression in normal breast epithelium and primary invasive ductal breast carcinoma (IDC) from 177 patients [30] and found that nuclear MBP-1 was found in almost all the normal tissues while its expression was retained in only 35% of the tumors. Statistically significant associations were observed among the nuclear expression of MBP-1 and ErbB2 status, Ki-67 expression, node status and tumor grade. Furthermore, the loss of MBP-1 expression makes the recurrence-free survival of patients fall from 92% to 54% with IDC [30]. Ghosh et al. found that MBP-1 could be a potential gene therapeutic candidate against non-small cell lung cancer (NSCLC) growth [31]. The exogenous expression of MBP-1 in NSCLC cells inhibits cell proliferation and induces mitochondrial pore formation which does not allow accumulation of cytochrome C. MBP-1 also increases the causes of necrosis-like cell death in the NSCLC cell line, H1299 cells, including cellular densification with highly disorganized nuclei, numerous small and large cytoplasmic vacuoles, lipid droplets, extensive cell lysis with accompanying cellular debris and numerous swollen and internally disorganized mitochondria.

Prostate cancer is the second leading cause of cancer death in the United States, and the American Cancer Society estimated that about 11% of men will develop invasive prostate cancer in their lifetime [32]. The research indicated that MBP-1 suppresses the growth of prostate cancer cells by mediating the MAPK pathway [33]. The results are helpful for the diagnosis and treatment of prostate cancer, and MBP-1 is expected to be a promising therapeutic target. At the same time, by up-regulating the expression of miR29b, MBP-1 inhibits the synthesis of collagen and MMP-2 (Matrix Metalloproteinase-2) proteins, which weakens the invasive ability of one prostate cancer cell line, PC13 cells, but suppresses the growth of two other prostate cancer cell lines, DU145 and PC3 cells [34]. Knockdown of MBP-1 in a prostate cancer cell line (PC3-4.2) perturbs cell proliferation by inhibiting cyclin A and cyclin B1 expression [35].

In addition, recent studies have demonstrated the biological effects of MBP-1 on human esophageal cancer EC109 cells and osteosarcoma Saos-2 cells [36,37]. When the expression of MBP-1 is down-regulated, the proliferation ability and quantity of G0/G1 phase cells of EC109 cells is decreased; the proportion of apoptosis increases, the number of invasive cells is reduced and the expression of c-Myc, cyclin D1 and cyclin E proteins are also suppressed [36]. In addition, MBP-1 also has important biological roles in conditions other than cancer. Such an example may be seen in the influence of MBP-1 in HIV activity. The results of transient co-transfection assay showed that the carboxyl terminal half of MBP-1 (190~335 aa) can also inhibit the activity of the long terminal repeat promoter of human immunodeficiency virus type-1 (HIV-1) [38].

## 5. MBP-1 in Plant Growth and Development

The *ENO2* gene is also expressed in all the tissues in plants and plays an integral role in plant growth, development and abiotic stress responses. Compared with wild-type *Arabidopsis thaliana*, *eno2*-mutants are characterized by shorter primary principal roots and siliques, less lateral roots and pollen grains, significantly lower germination rates of pollen and shorter over ground parts [39,40]. Marina et al. also discovered that the knockout of the *ENO2* gene reduced lignin and increased salicylic acid contents, as well as altered fatty acid and soluble sugar composition [14]. The study showed that the *Arabidopsis* plants that overexpress *AtMBP-1* were shorter, displayed an increased number of flowers and had shorter siliques than the wild type (WT). Furthermore, the overexpression of AtMBP-1 delayed the flowering time and slightly increased the seed size [13]. However, when *Arabidopsis* plants that have grown 18 days in soil were exposed to ABA with different concentrations, the growth of WT plants was inhibited by ABA treatment; however, *AtMBP-1* over-expressing plants showed a dose-dependent increase in growth. The size and leaf shape of *AtMBP-1* over-expressing plants were similar to that of WT plants at the higher ABA concentration [13]. Simultaneously, MBP-1 can reduce the expression of *STZ/ZAT10* and the activity of STZ/ZAT10 which can hinder the plant’s growth is also inhibited when the *AtMBP-1* coding sequence is mutated [2,13]. These studies show that *STZ/ZAT10* responds to stresses by binding upstream of stress-inducible genes (e.g., *DREB1a* and *AREB4*), inhibiting their expression [41].

## 6. MBP-1 in Abiotic Stress Responses

As cancer cells grow and proliferate, they outgrow the oxygen and nutrients supplied by the bloodstream and eventually encounter tumor hypoxia. Under hypoxic conditions, cancer cells demonstrate the Warburg effect, which is an increasing reliance on anaerobic respiration to generate ATP for cellular energy and biosynthetic pathways [42]. Research has shown that α-enolase increases anaerobic metabolism which may play a part in hypoxic tolerance of tumors, characteristic of hypoxic stress proteins [6]. It has been demonstrated that the progression of tumors, along with poor survival rates, are correlated with overexpression of α-enolase at both protein and RNA levels [24,43]. Similarly, the expression of MBP-1 is also affected by the concentration of oxygen in tumor and non-tumorigenic cell lines, but remains at much lower endogenous levels than α-enolase [44].

Upstream of the MBP-1 coding sequence, a hypoxia-response element (HRE) may bind to hypoxia-inducible factor 1α (HIF1α) to regulate α-ENO/MBP-1 expression in normal and cancer cells. In BEAS-2B cells, non-cancerous bronchial endothelial cells, MBP-1 mRNA levels were down-regulated under hypoxic conditions although endogenous MBP-1 protein levels remained undetectable under normal and hypoxic conditions [22]. However, MBP-1 protein expression levels were down-regulated under hypoxic conditions in MCF-7 cells, which are breast cancer cells [45]. The discrepancy between MBP-1 expression levels in non-cancerous BEAS-2B and cancerous MCF-7 under hypoxic conditions, may be due to distinct features of the tumor microenvironment. In addition, physiological level and high concentrations of sugar can lead to decreased expression of MBP-1, thus promoting cell proliferation and the production of lactic acid. However, at low glucose concentrations (1 nM), the expression of MBP-1 remains increased within 48 h [46]. Therefore, Maranto et al. thought the ability for transformed cells to survive low glucose and hypoxic conditions may stem from loss of MBP-1 function. [44].

The stress state of the endoplasmic reticulum can also promote the expression of MBP-1 as does glycolysis inhibitor 2-deoxyglucose and thapsigargin [44]. In *Arabidopsis thaliana*, the germination rate of seeds from the MBP-1 overexpression line can be inhibited by ABA, NaCl, mannitol and polyethylene glycol [14]. Therefore, MBP-1 plays an important role in response to abiotic stresses, whether it is in plants or cancer cells; however, the responding mechanism of MBP-1 to abiotic stresses still need further investigation.

## 7. Regulatory Network of MBP-1

**Figure 3:**
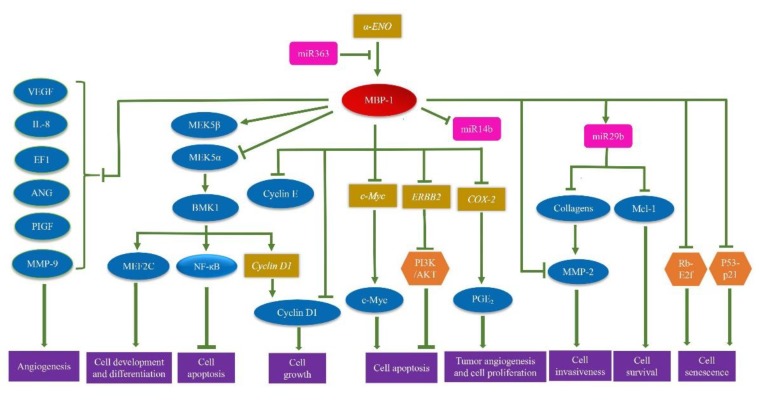
The regulatory network of MBP-1. Blue oval represents proteins; yellow rectangle represents genes; pink rectangle represents microRNA; orange rhombus represents signal pathway. Sharp arrow represents promotion of expression and blunt arrow represents inhibition of expression.

**Figure 4:**
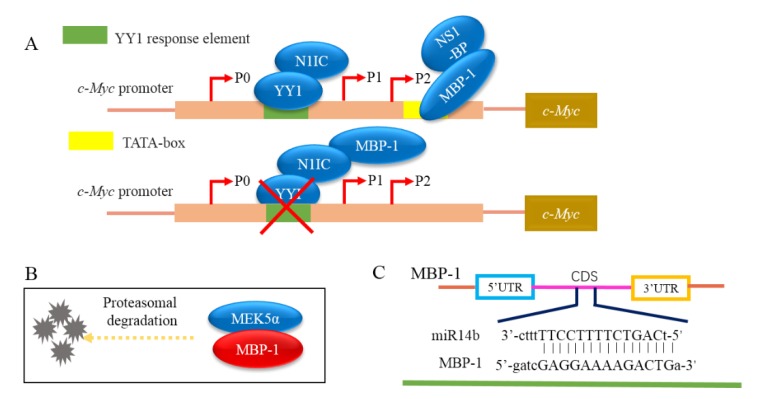
The schematic diagram of interactions between MBP-1 and proteins or miRNA. (**A**) The binding of NS1-BP with MBP-1 increases the inhibitory effect of MBP-1 on c-Myc promoter activity; the combination of MBP-1 and NI1C/YY1 complex attenuates the binding ability of NI1C/YY1 complex to c-Myc promoter. The red cross represents that the N1IC/YY1 complex doesn’t bind to the YY1 response element. (**B**) MBP-1 destabilizes MEK5α. (**C**) The binding sequence of MBP-1 and miR14b.

Three target genes of MBP-1, including *c-Myc* [17], *COX2* [27] and *ERBB2* [47], have been identified thus far. MBP-1 can inhibit the expression of *c-Myc* by binding to the P2 promoter of *c-Myc* and also inhibit the expression of *COX-2* and *ERBB2*. As shown in Figure 3, MBP-1 can affect the regulation of PI3K/AKT pathway by inhibiting the expression of the *ERBB2* gene in the breast cancer cells (SKBr3 cell line), thus suppressing cell apoptosis [47]. It also affects the Rb-E2f and p53-p21 signaling pathway, thereby inhibiting cell senescence in human foreskin fibroblasts and prostate cancer cells [48,49]. Moreover, MBP-1 down-regulates the expression of the *MEK5* gene and can interact with MEK5α protein to promote the degradation of MEK5α protein by proteasome pathways in prostate cancer cells (Figure 4B). Because MEK5α mediates the phosphorylation of BMK1 and leads to its activation, MBP-1 also inhibits the activation of MEK5/BMK1-mediated MEF2C, NK-κB and Cyclin D1 [33]. These results attenuated cell growth, development, differentiation and inhibited cell apoptosis. The research has also illustrated that MBP-1 suppresses the activity of endothelin-1 (ET-1), angiogenin (ANG), interleukin-8 (IL-8), MMP-9, placental growth factor (PGF) and vascular endothelial growth factor (VEGF), which all accelerate cell apoptosis in breast cancer cells (MCF-7) [50]. By interacting with N1IC which is the Notch1 receptor intracellular domain, MBP-1 can inhibit the binding of the N1IC/YY1 complex to the YY1 response element on the *c-Myc* promoter, thereby eliminating the promotion of *c-Myc* expression (Figure 4A) [51]. In vertebrates, the Notch signal pathway is involved in the control of several cellular functions, including cell fate decision, proliferation, differentiation, apoptosis, and tumorigenesis. The combination of MBP-1 and N1IC can also repress the binding of the N1IC/YY1 complex to the YY1 response element on the c-Myc promoter, thereby eliminating the promotion effect of the N1IC/YY1 complex on the activity of the *c-Myc* promoter [51]. However, after MBP-1 is combined with NS1-BP, NS1-BP enhances the ability of MBP-1 to inhibit the activity of the c-Myc promoter [52].

MicroRNAs (miRNA), deriving from distinct hairpin precursors, are ∼22-nucleotide (nt) endogenous RNAs found in both plants and animals [53]. When combined with an argonaute protein containing silencing complex, allows for post-transcriptional repression or a protein by repressing at the translational level, destabilizing the transcript and sometimes a combination of both methods [54]. The expression of MBP-1 can also be regulated by miRNA. Studies have shown that exogenous overexpression of miRNA363 reduced MBP-1/α-ENO protein levels and miR-365-binding site located at 208–216 nt in the DNA sequence of MBP-1/α-ENO [55]. In addition, miR14b and miR29b interact with MBP-1. Among them, MBP-1 inhibits the expression of miR14b binding site which is 5’-GAGGAAAAGACTG-3’ (Figure 4C), but up-regulates the expression of miR29b. Interestingly, induction of miR-29b can be achieved along with inhibition of MMP-2 activation by MBP-1; miR-29b results in a cascade of repressed expression of *MMP-2*, *MCL-1* and multiple collagens. In cells, Mcl-1 improves the rate of cellular survival and MMP-2 promotes cell invasiveness [34].

**Figure 5:**
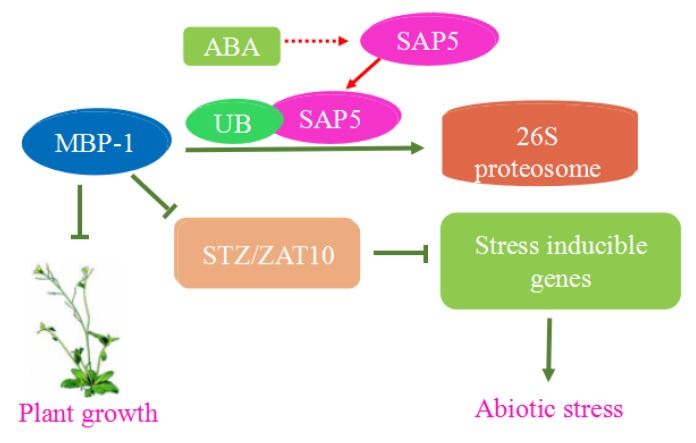
Hypothetical model of the action of ABA, AtSAP5 and AtMBP-1 (refer to Miyoung et al., 2013 [13]). MBP-1 suppresses *STZ/ZAT10* gene expression which can respond to ABA and plant growth. ABA increases the expression of AtSAP5 which can promote the degradation of AtMBP-1 by the ubiquitin-dependent proteasome pathway. Sharp arrow represents the function of promotion and blunt arrow represents the function of inhibition. The solid line indicates that the interaction between the two is clear and the dotted line indicates that the interaction between the two is not clear.

In *Arabidopsis thaliana*, the expression of MBP-1 is modulated by ABA and AtSAP5. ABA increases the content of AtSAP5 in plants, and AtSAP5 through ubiquitination can interact with AtMBP-1, thus promoting the degradation of AtMBP-1 [13]. In addition, AtMBP-1 represses the expression of the *STZ/ZAT10* gene which binds to upstream of stress inducible genes in *Arabidopsis*, including the master stress-responsive transcriptional regulatory genes *DREB1a* (*CBF3*) and *AREB2* (*abf4*), and suppresses their expression [41,56]. The reciprocally regulatory relationship among them is shown in Figure 5.

## 8. The Developing Potential of MBP-1 in Pharmaceuticals

As the target gene of MBP-1, *c-Myc* is a highly pleiotropic transcription factor known to regulate cell cycle progression, apoptosis, and cellular transformation and it controls nearly 15% of the expression of all genes in vertebrates [57,58]. The expression of *c-Myc* is detected and is related to poor clinical outcome, aggressive metastatic phenotype, and increased tendency for relapse in 20% of all human cancers [59,60]. Strong evidence from epidemiological data support the idea that Aspirin reduces the risk of cancers of the colon, breast, prostate, lung, and skin [61,62,63,64] and its use lowers risks and increases patient survival after colorectal cancer diagnosis [56]. The experiments indicated that aspirin and its primary metabolic salicylic acid down-regulate cyclin A2/CDK2 proteins and c-Myc levels both transcriptional and post-transcription levels in multiple cancer cell lines, such as HCT-116, SW480, and NCI-H226 [65,66]. Furthermore, aspirin consists of acetyl and salicylate groups; the later contributes to its anti-inflammatory properties via inhibition of NF-κB [67], whereas the acetyl group deactivates cyclooxygenases (COX) through acetylation of serine residues [68]. MBP-1 has the same function as aspirin and salicylic acid to some extent. The mutation of *ENO2*, which alternatively translates MBP-1 protein, increases the content of salicylic acid in *Arabidopsis thaliana* [14]. Thus, MBP-1 protein is expected to be developed as a new pharmaceutical ingredient for cancer treatment and prevention. Salouti et al. have found the synergistic effect on the healing of infected wounds caused by *Staphylococcus aureus* by using a plant peptide MBP-1 and silver nanoparticles combination in a mouse model [69]. Additionally, the apoptosis induced by etoposide, doxorubicin, and cisplatin which are currently used in cancer therapy can be enhanced by c-Myc overexpression in NIH3T3 mouse fibroblasts, TGR-1, HO15.19, and HOmyc3 Rat1 cell lines [70] Therefore, it is worth studying whether these drugs can target MBP-1. However, developing drugs to target MBP-1 will be a challenge because it lacks enzyme activity and is a transcription factor as with c-Myc [71].

## 9. Conclusions

In conclusion, MBP-1 is expressed in a wide range of tissues and cell types in vertebrates and plants, and some miRNAs, such as miRNA363, miRNA14b and miRNA25b, can modulate its expression levels. By interacting with genes and proteins, MBP-1 has an important impact on the growth, reproduction and invasion of cancer cells and also plays an important role in plant growth and development.

In summary, tremendous progress has been made in understanding the cellular functions of MBP-1 although more efforts are needed to understand the functional mechanism of MBP-1. Both MBP-1 and ENO2/α-ENO are translated from the identical coding exons within the same gene but with alternative start codons, and the functional details of both proteins remain elusive, especially in terms of affecting plant growth and development or responding to stress. The role of MBP-1 as a negative regulatory transcription factor has been well described; however, its target genes and binding partners remain largely unknown. MBP-1 localizes in the nucleus, but it does not contain a nuclear localization signal (NLS), indicating a different subcellular localization mechanism between ENO2/α-ENO and MBP-1. Future advances in these research areas will help us better understand the cellular and molecular mechanism of both ENO2/α-ENO and MBP-1 proteins, which could provide new foundation for medical and agricultural applications.

## Figures and Tables

**Table 1:** The effect of MBP-1 on cancer cell lines.

Cancer	Cell Line	Influence
Breast cancer	MCF-7	Inhibit anchorage-independent growth
	EO771	Inhibit growth and matrix metalloproteinase expression
	MDA-MB-231	Suppress the tumorigenicity
Esophageal cancer	EC109	Inhibit proliferation, apoptosis and invasion
Gastric cancer	SC-M1	Inhibit proliferation, colony formation, migration and invasion
	SGC-7901	Inhibit cell proliferation
Lung cancer	H1299	Inhibit proliferation and growth, induce necrosis-like cell death and mitochondrial dysfunction
Osteosarcoma	Saos-2	Inhibit cell proliferation by decreasing the expression levels of cyclin D1 and cyclin E1 protein
Prostate cancer	PC13	Inhibit cell growth
	DU145	Inhibit cell growth
	PC3-4.2	Delay cell cycle progression

## Supplementary Materials

Supplementary materials can be found at http://www.mdpi.com/1422-0067/19/12/3868/s1.
