# Supplementary material for: The Biological Significance and Regulatory Mechanism of c-Myc Binding Protein 1 (MBP-1)
Source: Int J Mol Sci. 2018 Dec 4;19(12):3868. doi: 10.3390/ijms19123868 (PMC6320933; doi:10.3390/ijms19123868)
Supplement: Supplementary file 1 [file ijms-19-03868-s001.pdf]

Supplementary materials

α-ENO/MBP-1:

UniProt accession number: **P06733**

The results of Phyre2:

α-ENO

PDB accession numbers: **3B97**

The results of Phyre2:

Input:

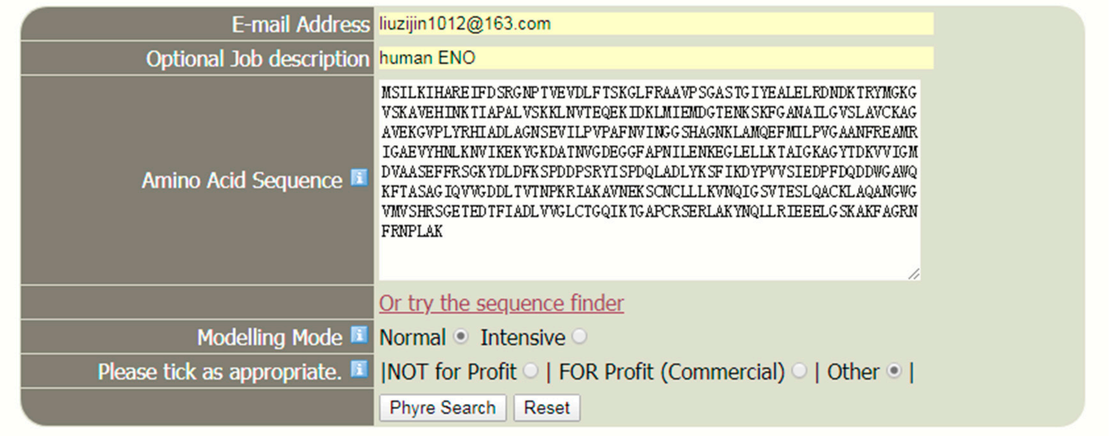

MSILKIHAREIFDSRGNPTVEVDLFTSKGLFRAAVPSGASTGIYEALELRDNDKTRYMG
KGVSKAVEHINKTIAPALVSKKLNVTEQEKIDKLMIEMDGTENKSKFGANAILGVSLAV
CKAGAVEKGVPLYRHIADLAGNSEVILPVPAFNVINGGSHAGNKLAMQEFMILPVGA
ANFREAMRIGAEVYHNLKNVIKEKYGKDATNVGDEGGFAPNILENKEGLELLKTAIG
KAGYTDKVVIGMDVAASEFFRSGKYDLDFKSPDDPSRYISPDQLADLYKSFIKDYPVVSI
EDPFDQDDWGAWQKFTASAGIQVVGDDLTVTNPKRIAKAVNEKSCNCLLLKVNQIGS
VTESLQACKLAQANGWGVMVSHRSGETEDTFIADLVVGLCTGQIKTGAPCRSERLAK
YNQLLRIEEELGSKAKFAGRNFRNPLAK

Output:

Confidence in the model: 100.0%

% identity: 84%

Template Information:

PDB Molecule: gamma enolase;

PDB Title: fluoride inhibition of enolase: crystal structure of the2 inhibitory complex

Sequence Alignment as follow:

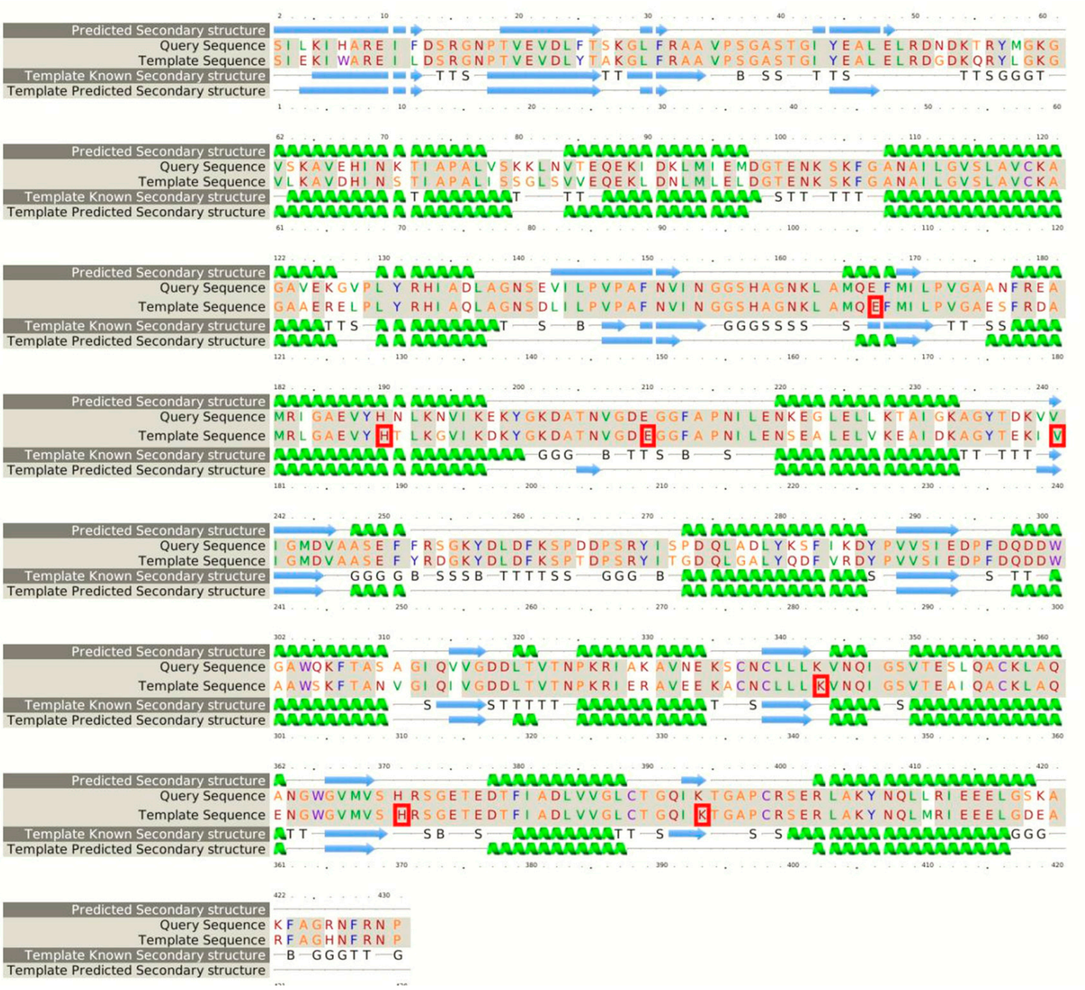

MBP-1:

PDB accession numbers: **not searched**

The results of Phyre2:

Input:

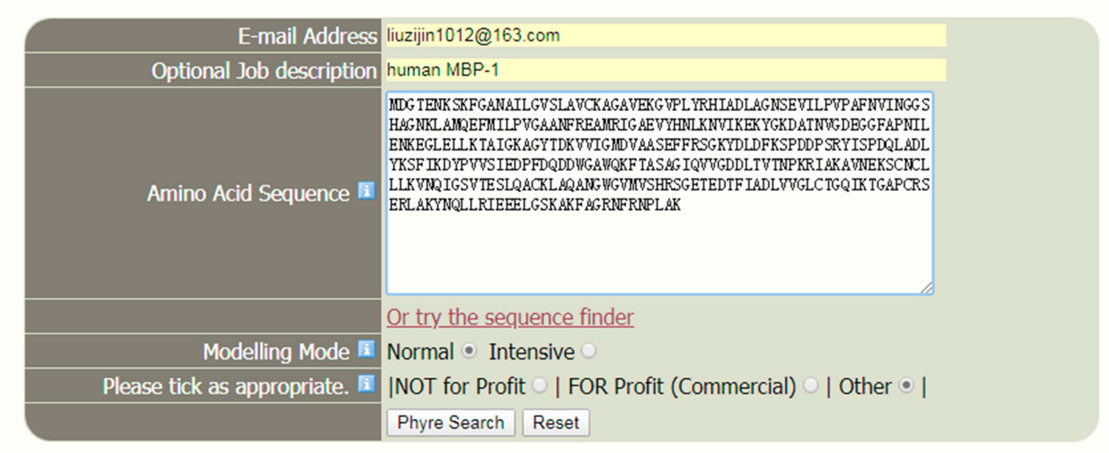

MDGTENKSKFGANAILGVSLAVCKAGAVEKGVPLYRHIADLAGNSEVILPVPAFNVIN
GGSHAGNKLAMQEFMILPVGAANFREAMRIGAEVYHNLKNVIKEKYGKDATNVGD
EGGFAPNILENKEGLELLKTAIGKAGYTDKVVIGMDVAASEFFRSGKYDLDFKSPDDPS
RYISPDQLADLYKSFIKDYPVVSIEDPFDQDDWGAWQKFTASAGIQVVGDDLTVTNPK
RIAKAVNEKSCNCLLLKVNQIGSVTESLQACKLAQANGWGVMVSHRSGETEDTFIAD
LVVGLCTGQIKTGAPCRSERLAKYNQLLRIEEELGSKAKFAGRNFRNPLAK

Output:

Confidence in the model: 100.0%

% identity: 85%

Template Information:

PDB Molecule:gamma enolase

PDB Title: fluoride inhibition of enolase: crystal structure of the2 inhibitory complex

Sequence Alignment as follow:

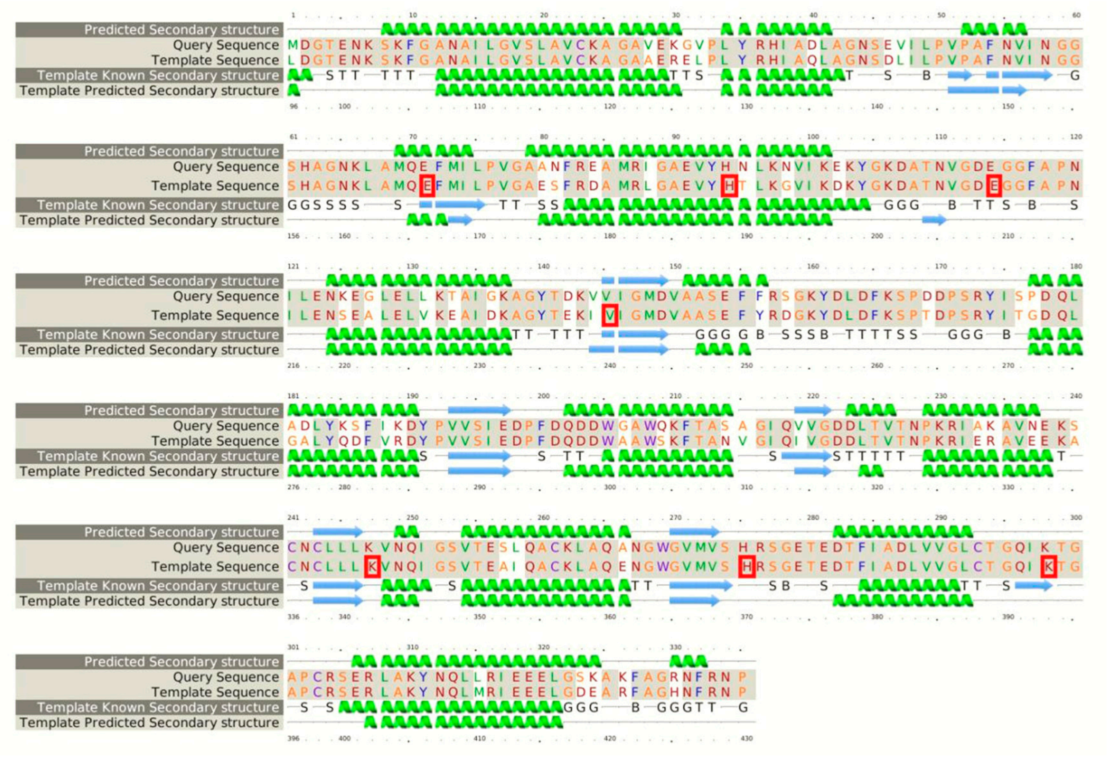
Predicted Secondary structure
Query Sequence
Template Sequence
Template Known Secondary structure
Template Predicted Secondary structure

AtENO2/AtMBP-1:

UniProt accession number: **P25696**

AtENO2:

PDB accession numbers: **not searched**

The results of Phyre2:

Input:

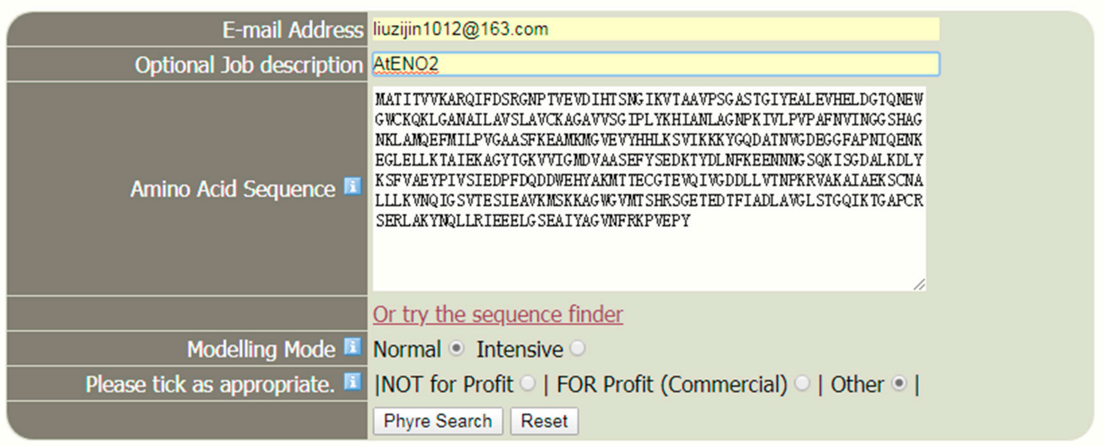

MATITVVKARQIFDSRGNPTVEVDIHTSNGIKVTAAVPSGASTGIYEALEVHELDGTQN
EWGWCKQKLGANAILAVSLAVCKAGAVVSGIPLYKHIANLAGNPKIVLPVPAFNVIN
GGSHAGNKLAMQEFMILPVGAASFKEAMKMGVEVYHHLKSVIKKKYGQDATNVGD
EGGFAPNIQENKEGLELLKTAIEKAGYTGKVVIGMDVAASEFYSEDKTYDLNFKEENN
NGSQKISGDALKDLYKSFVAEYPIVSIEDPFDQDDWEHYAKMTTECGTEVQIVGDDLLV
TNPKRVAKAIAEKSCNALLLKVNQIGSVTESIEAVKMSKKAGWGVMTSHRSGETEDTF
IADLAVGLSTGQIKTGAPCRSERLAKYNQLLRIEEELGSEAIYAGVNFRKPVEPY

Output:

Confidence in the model: 100.0%

% identity: 60%

Template Information:

PDB Molecule: enolase

PDB Title: 2.75 angstrom crystal structure of enolase 1 from toxoplasma gondii

Sequence Alignment as follow:

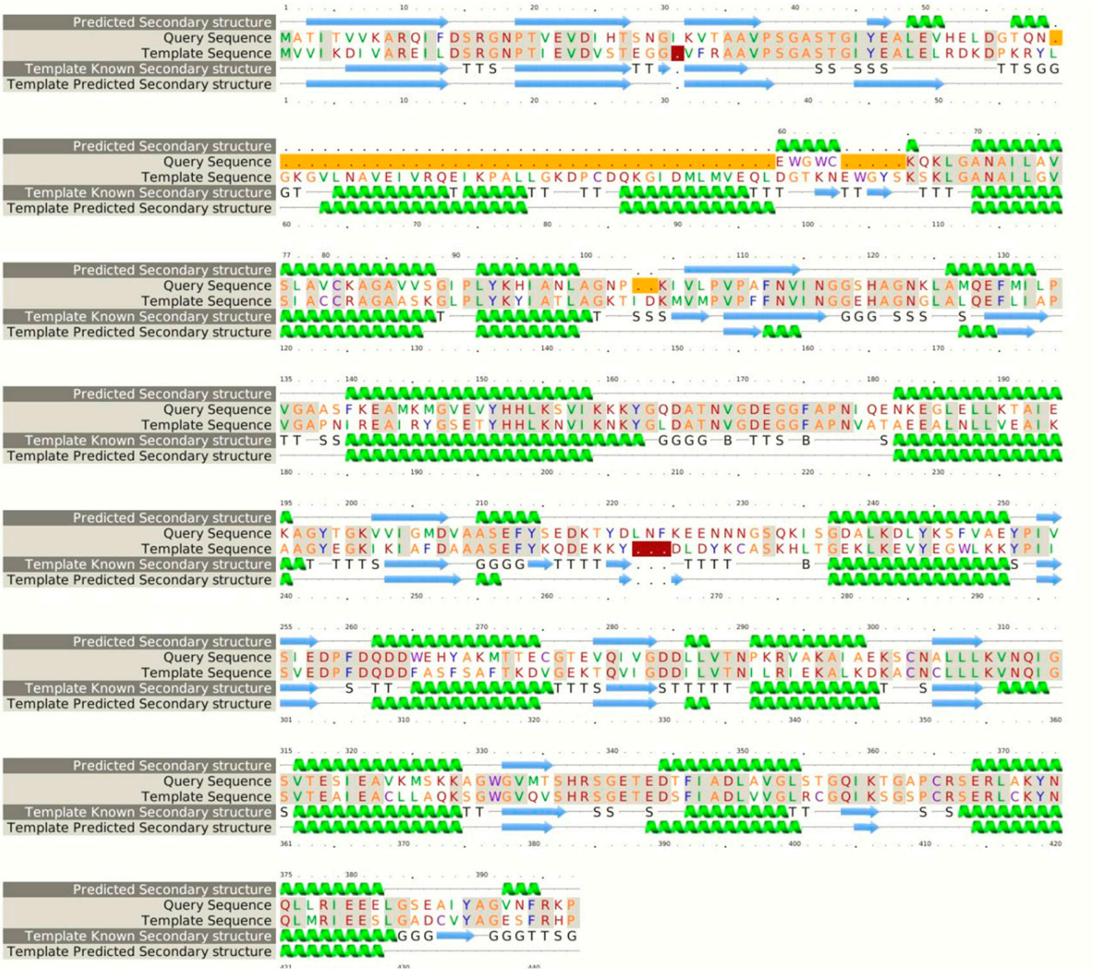
Predicted Secondary structure
Query Sequence MATITVVKARQIFDSRGNPTVEVDIHTSNGIKVTAAVPSGASTGIYEALEVHELDGTQN
Template Sequence MVVIKDIVAREILDSRGNPTIEVDVSTEGG.VFRAAVPSGASTGIYEALELRDKDPKRYL
Template Known Secondary structure
Template Predicted Secondary structure
Query Sequence EWGWC KQKLGANAILAV
Template Sequence GKGVLNAVEIVRQEIKPALLGKDPCDQKGIDMLMVEQLDGTKNEWGYSKSKLGANAILGV
Query Sequence SLAVCKAGAVVSGIPLYKHIANLAGNP..KIVLPVPAFNVINGGSHAGNKLAMQEFMILP
Template Sequence SIACCRAGAASKGLPLYKYIATLAGKTIDKMVMPVPFFNVINGGEHAGNGLALQEFLIAP
Query Sequence VGAASFKEAMKMGVEVYHHLKSVIKKKYGQDATNVGDEGGFAPNIQENKEGLELLKTAIE
Template Sequence VGAPNIREAIRYGSETYHHLKNVIKNKYGLDATNVGDEGGFAPNVATAEEALNLLVEAIK
Query Sequence KAGYTGKVVIGMDVAASEFYSEDKTYDLNFKEENNNGSQKISGDALKDLYKSFVAEYPIV
Template Sequence AAGYEGKIKIAFDAAASEFYKQDEKKY...DLDYKCASKHLTGEKLKEVYEGWLKKYPII
Query Sequence SIEDPFDQDDWEHYAKMTTECGTEVQIVGDDLLVTNPKRVAKAIAEKSCNALLLKVNQIG
Template Sequence SVEDPFDQDDFASFSAFTKDVGEKTQVIGDDILVTNILRIEKALKDKACNCLLLKVNQIG
Query Sequence SVTESIEAVKMSKKAGWGVMTSHRSGETEDTFIADLAVGLSTGQIKTGAPCRSERLAKYN
Template Sequence SVTEAIEACLLAQKSGWGVQVSHRSGETEDSFIADLVVGLRCGQIKSGSPCRSERLCKYN
Query Sequence QLLRIEEELGSEAIYAGVNFRKP
Template Sequence QLMRIEESLGADCVYAGESFRHP

AtMBP-1:

PDB accession numbers: **not searched**

The results of Phyre2:

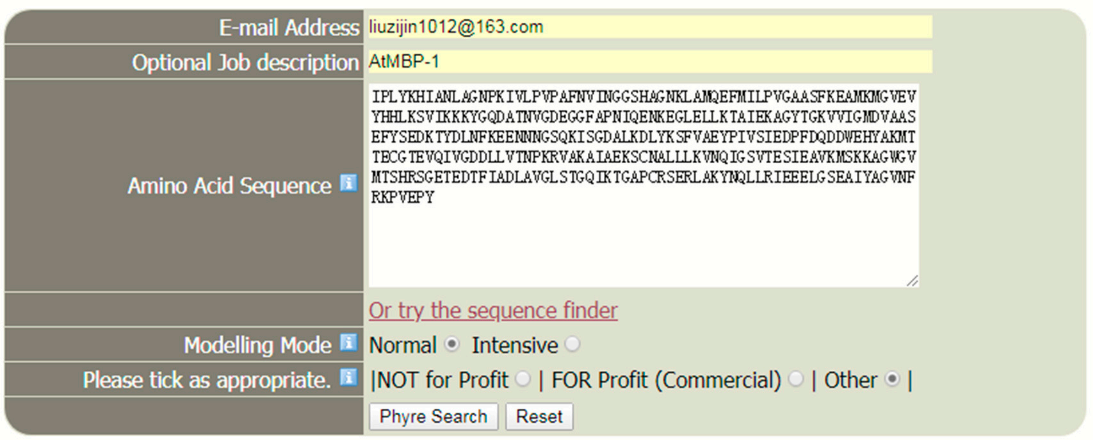

IPLYKHIANLAGNPKIVLPVPAFNVINGGSHAGNKLAMQEFMILPVGAASFKEAMKM
GVEVYHHLKSVIKKKYGQDATNVGDEGGFAPNIQENKEGLELLKTAIEKAGYTGKVVI
GMDVAASEFYSEDKTYDLNFKEENNNGSQKISGDALKDLYKSFVAEYPIVSIEDPFDQD
DWEHYAKMTTECGTEVQIVGDDLLVTNPKRVAKAIAEKSCNALLLKVNQIGSVTESIE
AVKMSKKAGWGVMTSHRSGETEDTFIADLAVGLSTGQIKTGAPCRSERLAKYNQLLRI
EEELGSEAIYAGVNFRKPVEPY

Output:

Confidence in the model: 100.0%

% identity: 74%

Template Information:

PDB Molecule: Enolase C-terminal domain-like

PDB Title: Crystal structure of enolase1

Sequence Alignment as follow:

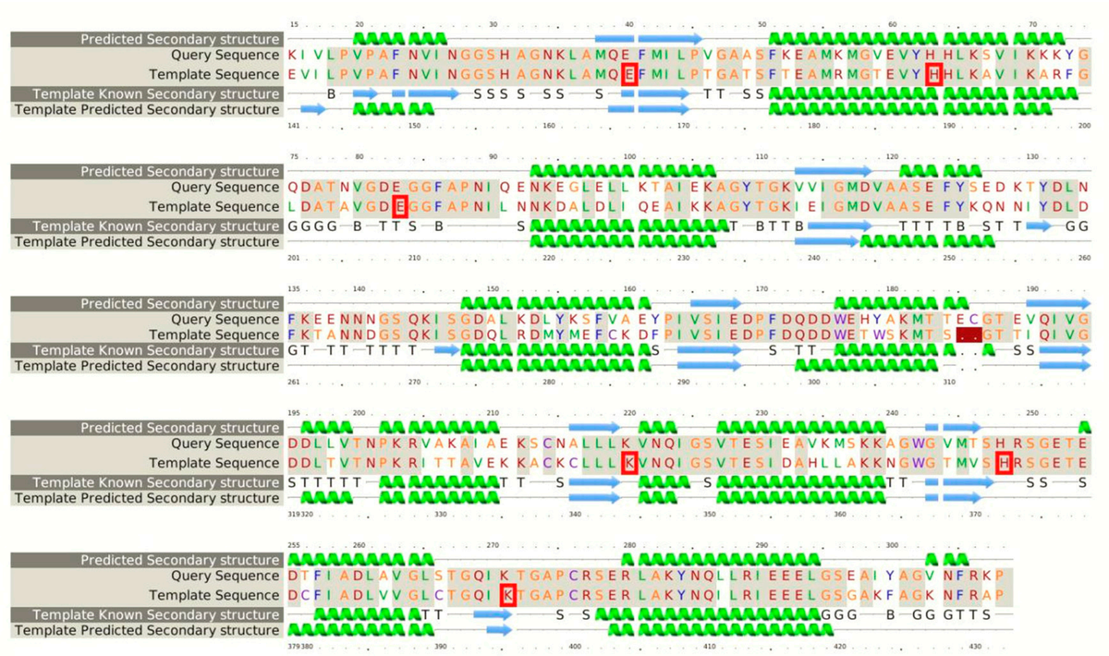
Predicted Secondary structure
Query Sequence
Template Sequence
Template Known Secondary structure
Template Predicted Secondary structure
